# Directional migration of adult hematopoeitic progenitors to C6 glioma *in vitro*

**DOI:** 10.3892/ol.2015.2952

**Published:** 2015-02-10

**Authors:** IGOR STEPANOVICH BRYUKHOVETSKIY, POLINA VIKTOROVNA MISCHENKO, ELENA VADIMOVNA TOLOK, SERGEI VICTOROVICH ZAITCEV, YURI STEPANOVICH KHOTIMCHENKO, ANDREI STEPANOVICH BRYUKHOVETSKIY

**Affiliations:** 1 Laboratory of Molecular and Cellular Neurobiology, School of Biomedicine, Far Eastern Federal University, Vladivostok 690091, Russia; 2 Laboratory of Pharmacology, A.V. Zhirmunski Institute of Marine Biology, Far Eastern Branch of the Russian Academy of Sciences, Vladivostok 690041, Russia; 3 NeuroVita Clinic of Interventional and Restorative Neurology and Therapy, Moscow 115478, Russia

**Keywords:** glioblastoma multiform, glioma C6, hematopoietic stem cells, neural stem cells

## Abstract

Multiform glioblastoma is the most common primary, highly invasive, malignant tumor of the central nervous system, with an extremely poor prognosis. The median survival of patients following surgical resection, radiation therapy and chemotherapy does not exceed 12–15 months and thus, novel approaches for the treatment of the disease are required. The phenomenon of the directed migration of stem cells in tumor tissue presents a novel approach for the development of technologies that facilitate the targeted delivery of drugs and other therapeutic agents to the tumor foci. Hematopoietic cluster of differentiation (CD)34^+^/CD133^+^ stem cells possess significant reparative potential and are inert with respect to normal neural tissue. The aim of the present study was to investigate the substantiation ability of adult hematopoietic progenitors to the directed migration of glioma cells. A C6 glioma cell line, a culture of hematopoietic CD34^+^/CD133^+^ stem cells and primary cultures of rat astrocytes and fibroblasts were used. The cells were co-cultured for five days. The results revealed the formation of cell shaft hematopoietic stem cells on the perimeter of the culture inserts containing the glioma culture. However, this was not observed in the wells with fibroblast and astrocyte cultures. The results indicated that hematopoietic stem cells exhibit a high potential for the directional migration of C6 glioma cells, which allows them to be considered as a promising cell line for the development of novel anticancer biomedical technologies and increases our understanding with regard to previously unclear aspects of glial tumor carcinogenesis.

## Introduction

Multiform glioblastoma is the most common primary, highly invasive, malignant tumor of the central nervous system, with an extremely poor prognosis. The median survival of patients after surgical resection, radiation therapy and chemotherapy does not exceed 12–15 months (1–3). The tumor is characterized by invasive growth, infiltration of the brain substance and high resistance to radiation and drug therapy, and thus, novel approaches are required for the treatment of the disease (4). In previous studies, the phenomenon of the directional migration of neural stem and progenitor cells in tumor tissue has been a focus, and has presented a significant opportunity for the targeted delivery of drug molecules to tumor-specific genes, antibodies and other therapeutic agents (5–9). In this way, the problem of selecting the optimal cell lines for transplantation is particularly important as the use of the patients own neural stem cells carries a high risk of neoplastic transformation (10–12).

We believe that hematopoietic CD34^+^/CD133^+^ stem cells may represent an alternative, as their collection and maintenance presents little difficulty, their use is not associated with ethical and legal restrictions and they have been successfully used for the treatment of cancer patients for >50 years (13).

In addition, studies have focused on neural stem and progenitor cells of the human brain as the most likely origin of malignant gliomas (5,14,15). The ability of hematopoietic progenitor cells and human adult mammals to migrate directionally in neoplastic foci identifies fundamentally novel aspects of carcinogenesis processes in the brain.

The aim of the present study was to obtain experimental evidence of the ability of hematopoietic progenitors of adult mammalian cells to cause the directed migration of rat glioma C6 lines when co-cultured. The study was approved by the ethics committee of the Far Eastern Federal University School of Biomedicine (Vladivostok, Russia), from December 14, 2012.

## Materials and methods

### Hematopoietic stem cells of rats

Rat hematopoietic stem cells were provided by the National Institute for Regenerative Medicine (Moscow, Russia). The phenotype and purity of the cell population was characterized by a FACScan flow cytometer (Becton-Dickinson, Franklin Lakes, NJ, USA) using anti-CD34/anti-CD133 fluorescein isothiocyanate-conjugated monoclonal antibodies. Prior to co-culture, the cells were stained with a fluorescent marker, the Vybrant CFDA SE Cell Tracer (V12883; Life Technologies, Grand Island, NY, USA), according to the manufacturer’s instructions.

### C6 glioma culture

The rat C6 glioma cell line has been used in previous studies as it exhibits similar characteristics to human gliblastoma cells, such as invasive growth and atypical nuclei (16,17). The C6 glioma line was provided by the School of Biomedicine, Far Eastern Federal University for the experiment. An aliquot containing 1×10^6^ tumor cells was thawed for 5 min at 37°C and washed free of dimethyl sulfoxide (Sigma-Aldrich, St. Louis, MO, USA), using Dulbecco’s modified Eagle’s medium (DMEM; Gibco Life Technologies, Carlsbad, CA, USA) containing 10% fetal bovine serum (FBS; Life Technologies) and 100X antibiotic-antimycotic (10,000 U/ml; Life Technologies). The cells were precipitated by centrifugation (120 × g for 3 min), fresh medium (DMEM) was added and then the cells were seeded into 50-ml culture flasks. Cultivation was continued until monolayer formation. Next, the cells were detached by enzymatic dissociation (0.05% trypsin-EDTA; MP Biomedicals, Santa Ana, CA, USA; 1:4; 10 min; 37°C), and after centrifugation (120 × g for 3 min) the supernatant was discarded and the cells were resuspended in fresh medium. Tumorigenicity was analyzed following the implantation of 0.5×10^6^ C6 glioma cells into the brain of adult rats using a stereotaxic device (Model 900 Small Animal Stereotaxic Instrument; David Kopf Instruments, Tujunga, CA, USA) as described previously (5).

### Isolation of primary cultures of rat fibroblasts

Primary culture fibroblasts were obtained from 12-day-old rat embryos (n=26). Female rats were anesthetized with ether, and disinfected with alcohol. Under sterile conditions, the abdomen was dissected and the uterine horns of the embryos were removed and placed in Hank’s solution (Life Technologies) supplemented with 100X antibiotic-antimycotic (Life Technologies). The head, limbs and internal organs were removed from the embryos. The remaining mass was separated into small sections using scissors and subjected to enzymatic dissociation with 0.25% trypsin for 40 min at 37°C. Cells were precipitated by centrifugation (1,000 × g for 5 min). The pellet was resuspended in complete medium (90% DMEM/F12, 10% HEPES, 10% FBS, 2 mM L-glutamine, 0.8% glucose, 0.2 U/ml insulin and 10,000 U/ml antibiotic-antimycotic; Life Technologies). The rats were provided by the Far Eastern Federal University.

### Isolation of primary cultures of rat astrocytes

A total of 28 newborn female white rats (weight, 3–5 g) were used for the experiment. The rats were provided by the Far Eastern Federal University. Under sterile conditions, the rats were anesthetized, the head was separated from the body, the skull was dissected and the brain was removed and placed in a petri dish containing Hank’s solution. Next, the cerebral cortex was isolated and purified from its vascular membranes, separated into small pieces using scissors, washed twice with Hank’s solution, and subjected to enzymatic dissociation with 0.05% trypsin-EDTA for 10 min at 37°C. Enzymatic dissociation was stopped by adding 5% FBS (Hank’s solution), washing twice and subjecting the sections to mechanical dissociation using the fused end of a Pasteur pipette. The cell suspension was precipitated by centrifugation (120 × g for 3 min) and resuspended in complete medium [DMEM/F12 (90%), fetal calf serum (10%), L-glutamine (2 mM), glucose (0.8%), insulin (0.2 U/ml) HEPES (25 mM), antibiotic-antimycotic (10,000 U/ml)].

### Establishment of co-cultures

**Figure 1:**
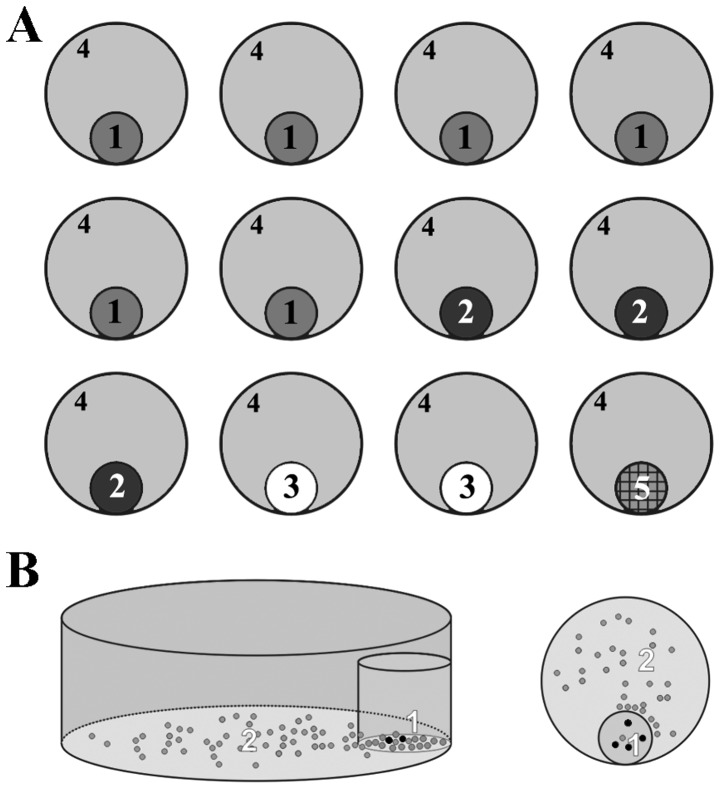
General scheme of the experiment. (A) Culture plates comprising (1) C6 glioma cells, (2) astrocytes, (3) fibroblasts, (4) hematopoietic stem cells and (5) an empty insert. (B) A culture insert with (1) C6 glioma cells co-culturing with (2) hematopoietic stem cells.

Culture inserts (diameter, 12 mm; pore size, 0.4 μm; EMD Millipore, Billerica, MA, USA), were used for the co-cultures. The bottom of a 12-well tissue culture plate was coated with polyethylenimine, and then with laminin (both Life Technologies). Next, culture inserts were immobilized using a drop of sterile paraffin, which was added to each well. A total of 0.5×10^6^ tumor cells and normal fibroblasts and astrocytes were added to the inside culture inserts, and one of the inserts was left empty (Fig. 1A). The plate with culture inserts was incubated for 24 h, then the bottom of the wells were plated with 0.25×10^6^ hematopoietic stem cells. Following incubation for 3 h, the non-adherent cells from the bottom of the wells were removed. The distribution of hematopoietic stem cells on the bottom of the wells was analyzed visually with a confocal laser scanning microscope (Zeiss LSM 710 META; Carl Zeiss, Jena, Germany) (Fig. 1B). Cell counts in the projection area of the membrane culture inserts were performed using Photo-Capt v.12.4 c 4 (Vilber Lourmat, Eberhardzell, Germany) between the first and fifth days of culture.

### Statistical analysis

Statistical analysis was performed using Statistica 6.0 software (StatSoft, Inc., Tulsa, OK, USA) and Microsoft Excel 2010 (Microsoft Corporation, Redmond, WA, USA).

## Results

### Hematopoietic stem cell culture

**Figure 2:**
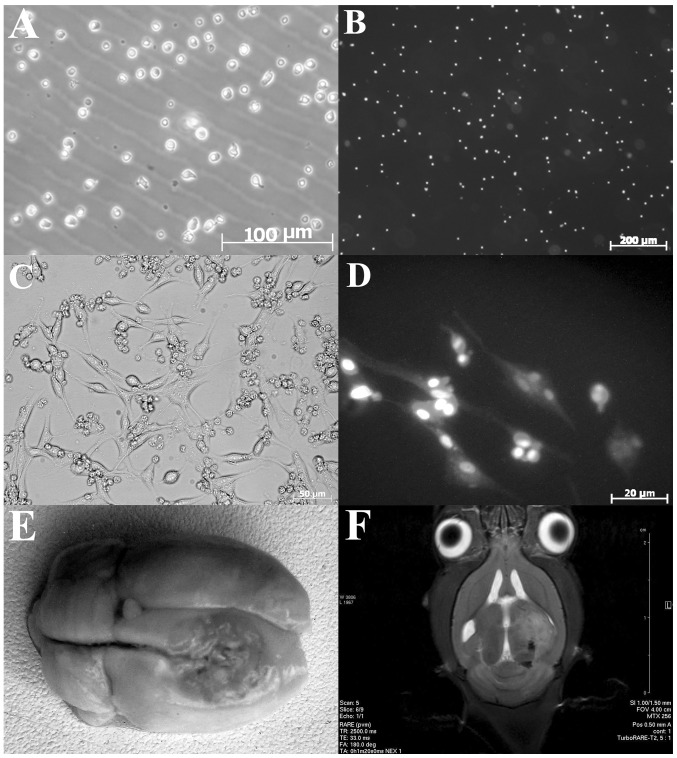
Characteristics of hematopoietic stem cells and neoplastic C6 glioma line. (A) Phase contrast microscopy of hematopoietic stem cells in culture (magnification, ×20). (B) Fluorescence microscopy of hematopoietic stem cells stained with Vybrant^®^ CFDA SE Cell Tracer directly prior to co-culture (magnification, ×10). (C) Inverted microscopy of neoplastic cells of rat C6 glioma. Cells of different shapes and sizes were observed actively flattened on the surface, forming numerous outgrowths on the third day of cultivation. Multinucleated cells were clearly visible (magnification, ×32). (D) Neoplastic C6 glioma cells stained using anti-glial fibrillary acidic protein monoclonal antibody, with nuclei counterstained using DAPI (magnification, ×100). (E) Rat brain preparation six days after the implantation of C6 glioma cells. (F) T2-weighted magnetic resonance imaging of the rat brain seven days after implantation of C6 glioma cells. Signs of compression of the ventricles of the brain edema and dislocation of medial brain structures were visible.

According to the data, the cytometry purity of the hematopoietic stem cell CD34^+^/CD133^+^ populations was 88.9%. The cells stained with Vybrant^®^ CFDA SE tracer exhibited a stable fluorescence, which was observed at low magnification (Fig. 2A and B).

### Primary culture of the C6 glioma line

C6 glioma cell culture presented a heterogeneous population of actively proliferating cells of different shapes and sizes (Fig. 2C). Elongated cells with outgrowths were clearly observed, and were surrounded by small, round cells. Subsequent to 24 h of cultivation, a number of cells with an astrocyte-like phenotype adhered to the bottom of the culture inserts, while other cells formed numerous outgrowths of different shapes and sizes. The cells were stained with monoclonal rabbit antibodies against glial fibrillary acidic protein (GFAP) (anti-GFAP antibody; ab33922; Abcam, Cambridge, UK). Microscopy at low magnification (with the Zeiss LSM 710 META confocal laser scanning microscope) and subsequent DAPI (D1306, Molecular Probes, Eugene, OR, USA) staining allowed the visualization of numerous neoplastic nuclei of various shapes and sizes (Fig. 2D). Stereotactic implantation of 3×10^4^ C6 glioma cells into the brains of the adult rats led to the development of tumors, which was accompanied by severe asthenia, a rapid decrease in the animal’s body weight, the development of severe edema and dislocation of brain structures, which was confirmed by morphological data and neuroimaging studies (Fig. 2D, E and F).

### Primary culture of rat fibroblast

**Figure 3:**
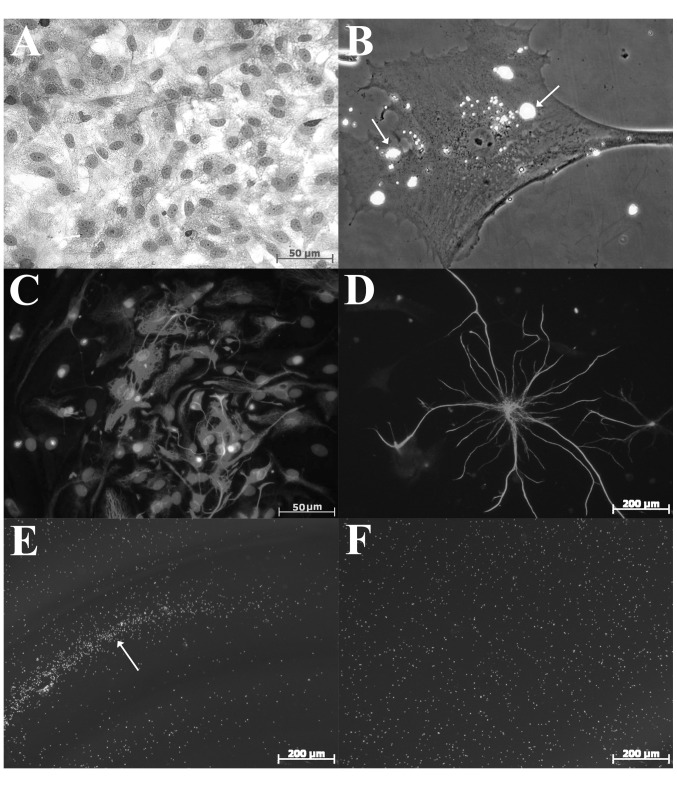
Characteristics of cell lines and control fibroblast cultures and astrocytes, and reaction of different cell lines to co-culturing with glioma cells in the experiment. (A) Rat fibroblasts (hematoxylin and eosin; magnification, ×40). (B) Rat fibroblasts. Phagocytosis of collagen immobilized on the surface of the fluorescent microparticles FluoSpheres^®^ Collagen I-Labeled Microspheres (magnification, ×630). (C) Rat astrocytes stained with anti-GFAP Mab and nuclei counterstained with DAPI (magnification, ×100). (D) Rat astrocyte stained with anti-GFAP Mab (magnification, ×200). (E) Formation of fluorescent cell shaft on the perimeter of culture inserts containing glioma cells, by hematopoietic stem cells stained with Vybrant^®^ CFDA SE Cell Tracer (magnification, ×10). (F) Co-culturing hematopoietic stem cells and rat fibroblasts. The formation of the cell shaft was absent (magnification, ×10). GFAP, glial fibrillary acidic protein; Mab, monoclonal antibody.

Hematoxylin and eosin staining of the rat fibroblasts revealed cells of different sizes and shapes with rounded cell nuclei (Fig. 3A). Functional analysis of the cells conducted using FluoSpheres^®^ Collagen I-Labeled Microspheres (F-20892; Life Technologies) according to the manufacturer’s instructions, revealed active phagocytosis of collagen particles (Fig. 3B). This culture was used as the control.

### Primary cultures of rat astrocytes

Cultured rat astrocytes were stained with DAPI and characterized using monoclonal antibodies against GFAP as a control (Fig. 3C and D).

### Migration of hematopoietic stem cells co-cultured with astrocytes, fibroblasts and C6 glioma

**Figure 4:**
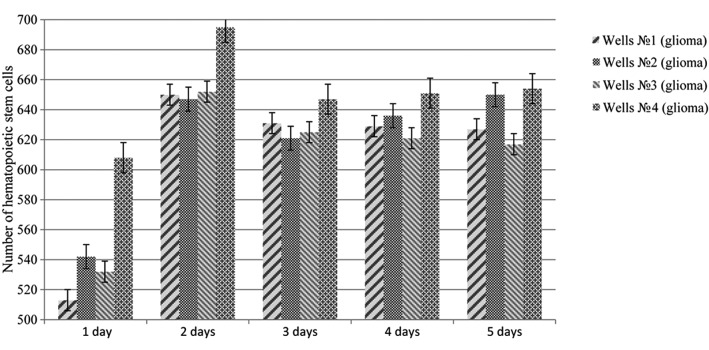
Distribution analysis showing the number of hematopoietic stem cells co-cultured with glioma between the first to the fifth days of the experiment.

**Figure 5:**
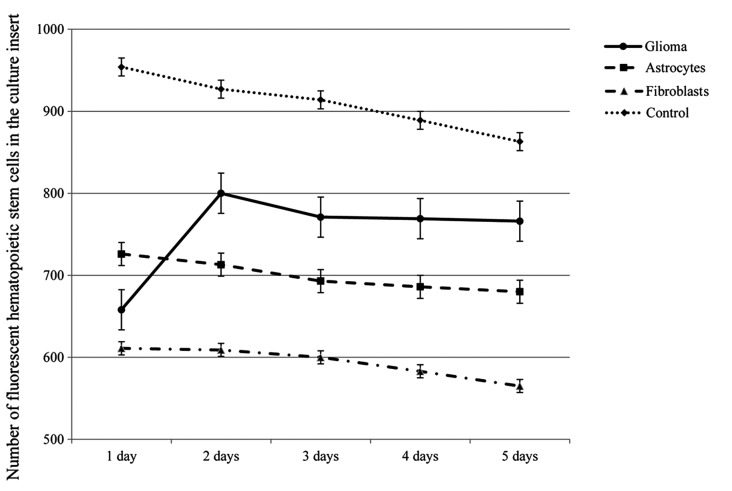
Comparative dynamics of the number of hematopoietic stem cells in culture inserts containing glioma C6 cells, astrocytes and fibroblasts between the first and fifth days of the experiment.

During the co-culture of the stem and tumor cells, the formation of the cell shaft consisting of hematopoietic stem cells, located on the perimeter of the culture insert, containing the glioma culture (Fig. 3E) was identified. This phenomenon, which was most significant on the second day, was identified in all co-cultures containing glioma cells, and absent in co-cultures containing fibroblasts and astrocytes (Fig. 3E and F). A fluorescent marker was used to count the cells, which showed a significant increase (P<0.05) in the number of adult hemopoietic progenitors in the field of the membrane in all co-cultures containing glioma cells between the second and fifth days of the experiment (Fig. 4). No significant differences in the number of hematopoietic stem cells in co-cultures with normal astrocytes and fibroblasts were identified (Fig. 5). We hypothesized that the directed migration of stem cells to co-cultured glioma caused the production of various chemoattractants and cytokines by neoplastic cells.

## Discussion

Identifying the phenomenon of the directed migration of stem cells to the area of injury, ischemic injury or neoplastic tissue was an important step in understanding the biology of carcinogenesis and regenerational processes in the brain of mammals and humans. It has been found that >79 cytokines, chemoattractants and growth factors, as well as ≥20 types of receptors, control the migration and homing of stem cells (18,19). The importance of stromal cell-derived factor-1α in this process and the active participation of stem cell factor, hepatocyte growth factor, vascular endothelial growth factor, monocyte chemoattractant protein-1, high-mobility group box 1, urokinase plasminogen activator, interleukin-6, the β1 and β2 integrins, L-selectin and other ligands has been investigated (20). The main source of chemoattractants, which attract stem cells, are damaged neurons, astrocytes, microglia and degenerating elements of the extracellular matrix that are released into the bloodstream. As can be observed in the present experiment, the primary source of cytokines is tumor tissue. The ability of neuroepithelial tumors to produce tenascin, fibronectin, laminin, various types of collagen and other biologically active molecules has been investigated previously (21). I*n vivo*, the main source of multipotent cell elements is the mature brain germinal zone. Tumors attract and actively recruit neural stem cells during the mutational process, stimulating the expression of the majority of oncogenes, and actively using their replication and migration potential (22).

Hematopoietic stem cells and adult progenitor cells of mammals and humans are significantly less involved in the mutational process than neural stem cells, as evidenced by the analysis of the proteomic profile. Furthermore, their high regenerative potential, in combination with their ability to maintain an association with normal nerve tissue allows their consideration as the most promising cell line for the treatment of the majority of neurological diseases and brain injuries (23,24).

In conclusion, the results of the present study indicate the pathotropism of mammalian hemopoietic stem cells to C6 glioma, which represents potential for their clinical application. However, the role of hematopoietic stem and progenitor cells in the carcinogenesis of malignant neoplasms of the brain requires further investigation.
